# A method for the extraction of microplastics from solid biowastes including biosolids, compost, and soil for analysis by µ-FTIR

**DOI:** 10.1016/j.mex.2024.102761

**Published:** 2024-05-17

**Authors:** Helena Ruffell, Olga Pantos, Brett Robinson, Sally Gaw

**Affiliations:** a School of Physical and Chemical Sciences, University of Canterbury, Christchurch, New Zealand; b Institute of Environmental Science and Research, Christchurch, New Zealand

**Keywords:** Microplastics, Biosolids, Compost, Soil, Wet peroxide oxidation, Density separation, µ-FTIR, Extraction methods, Extraction of microplastics from solid biowastes including biosolids, compost, and soil by a digestion with wet peroxide oxidation and density separation with sodium iodide in a sediment-microplastic isolation (SMI) unit

## Abstract

Few methods exist detailing the extraction of microplastics from organic matrices. A validated method for the successful extraction of microplastics from solid biowastes including biosolids, compost, and soil for spectroscopic analysis by micro-Fourier transform infrared spectroscopy (µ-FTIR) was developed. Solid dry biowastes were first digested with a wet peroxide oxidation (WPO) with iron (II) solution and 30% hydrogen peroxide followed by sequential density separations with ultra-pure water and 1.8 g cm^−3^ NaI in an optimised sediment-microplastic isolation (SMI) unit. The average recoveries for spiked microplastics were 92, 95 and 98% for bagged compost, biosolids, and soil, respectively. This method ensures a high microplastic recovery by first chemically disintegrating biowaste aggregates without employing destructive methods like milling and allows for successful density separations where the settled fraction is isolated off from the supernatant, allowing thorough rinsing of the equipment and thus a greater transferal of particles into the vacuum filtering device. Minimal processing steps reduce the instance of introducing contamination and particle loss.

• Digestion as a first step to disintegrate aggregates to release entrapped microplastics
• Density separation with SMI unit with the method adapted for biowastes
• Minimal steps to reduce contamination and particle loss

## Graphical abstract

**Figure:**
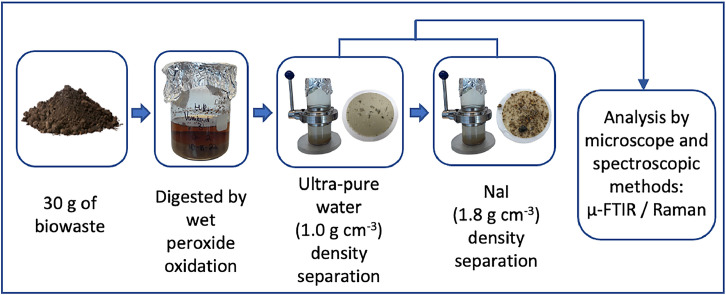

Specifications table

**Table:** 

Subject area:	Environmental Science
More specific subject area:	Microplastics
Name of your method:	Extraction of microplastics from solid biowastes including biosolids, compost, and soil by a digestion with wet peroxide oxidation and density separation with sodium iodide in a sediment-microplastic isolation (SMI) unit
Name and reference of original method:	*R.R. Hurley, A.L Lusher, M Olsen, L Nizzetto, Validation of a method for extracting microplastics from complex, organic-rich, environmental matrices, Environ. Sci. Technol. 52(13), (2018) 7409–7417.*https://doi.org/10.1021/acs.est.8b01517
Resource availability:	Original paper describing SMI unit: *R.*L. *Coppock, M Cole, P.K. Lindeque, A.M Queirós, T.S Galloway, A small-scale, portable method for extracting microplastics from marine sediments, Environ. Pollut. 230 (2017) 829–837.*https://doi.org/10.1016/j.envpol.2017.07.017

**Method details**

## Background

Microplastics in the aquatic environment have received much scientific attention, however, there is a lack of knowledge about microplastic contamination in the terrestrial environment, in part due to challenges with separating microplastics from solid matrices. Compared to aqueous samples where direct filtering of the sample onto a membrane can be employed, multiple processing steps are required to isolate microplastics from solid matrices. For the analysis by spectroscopic methods including Fourier-transform infrared spectroscopy (FTIR) and Raman, microplastics can be easily extracted from sediment and sand samples by a density separation without prior digestion of organic material [14]. Soil and other organic matrices generally require both a digestion to oxidise organic material to expose microplastics which may be embedded into aggregates, and a density separation to extract microplastics [6]. These methods actively aim to reduce the amount of organic material in the final sample which is usually vacuum filtered onto a membrane and may be subject to visual analysis first under stereomicroscope followed by spectroscopic methods.

This paper describes the combination and further optimisation of two previous methods. The first described the extraction of microplastics from sludge and soil samples with organic material oxidation by Fenton's reagent and sequential density separation with water and sodium iodide (1.8 g cm^−3^) to isolate microplastics [6]. The authors suggested there was no significant difference between the ordering of the method steps (i.e. organic matter removal first followed by density separations and vice-versa) [6]. We decided to first remove organic material to disintegrate aggregates by digestion to release potentially trapped microplastics followed by density separation. The use of digestion was selected instead of mechanical milling techniques which may fragment microplastics.

Many studies do not discuss the method of supernatant collection, but most often the supernatant is directly decanted from the sample beaker. Direct decanting of the supernatant was not successful in our trials. Spiked microplastics did not decant and instead adhered to the walls of the vessel, and settled particulate matter transferred into the filtering apparatus. An alternative method of separating the supernatant is centrifuging the digested solids with a high-density saturated salt solution to compact the solids into a pellet to aid in decanting the supernatant [3,12,16,[18], [19], [20]]. Centrifuging of samples may further fragment or compact microplastics into the solids pellet reducing recovery efficiency [4]. Additional methods for isolating the supernatant included syphoning with a glass pipette [5], and skimming with a spoon [1]. Radford et al. [15] used an ‘overflow’ method where briefly, excess saturated salt solution was poured into the beaker containing the settled density separation, causing the excess top layer to spill over into a larger surrounding collection dish, which was then collected and filtered. The authors reported microplastic recoveries of 59 – 84% using different reagents with this method. However, this method may agitate the settled fraction and cause excess residual solids to spill over, and introduce contamination by ambient microplastics. Some methods describe the use of separation funnels where organic material is drained first [[7], [8], [9],^11^,^17^] however this was not effective in trials in our laboratory as residual fine particulate material from the digested biosolids adhered to the glassware instead of being released when the valve was opened.

Coppock et al. [2] described an alternative separating unit, a sediment-microplastic isolation (SMI) unit where after the valve is closed, the top section of the unit is decanted by tipping into a filtering apparatus. This allows for the thorough rinsing of the top unit to allow an effective transfer of all material from the top of the unit, with no introduction of particulate material from the settled fraction. In the method by Coppock et al. [2], a saturated salt solution was directly added to a dried sediment sample in the SMI unit. However, in the method by Hurley et al. [6] the completed digest includes an aqueous fraction along with settled particulate material which makes the addition of a saturated salt solution challenging. An SMI unit has not previously been used with digests of complex organic matrices like soil, biosolids, or composts. We hypothesised that merging the methods from Hurley et al. [6] and Coppock et al. [2] together would optimise the ability to recover microplastics in the supernatant. Our improved method incorporates the SMI unit to separate the supernatant containing the extracted microplastics from the digested sample matrix. This paper also describes the modifications required to ensure a successful extraction of microplastics from solid matrices including biosolids, compost and soil following these two methods, along with results of spiked recoveries to demonstrate its effectiveness.

## Materials and methods

### Chemicals and materials

Analytical reagent grade acetone, iron (II) sulfate heptahydrate, sulfuric acid, and Decon 90 detergent were purchased from Thermofisher Scientific Inc. (Massachusetts, USA). Hydrogen peroxide (H_2_O_2_, 30%) was purchased from Jasol New Zealand Ltd. (Auckland, New Zealand). Sodium iodide (NaI) was purchased from Livestock Supplies New Zealand (Gore, New Zealand). Ultra-pure water (ASTM type I water, > 18.2 MΩ) was sourced from a Sartorius Arium® pro filtration system, fitted with a 0.45 µm filter (Sartorius AG, Göttingen, Germany). Whatman grade GF/C glass microfibre filters (1.2 µm pore, 47 mm diameter) were purchased from Merck KGaA (Darmstadt, Germany). Plastic reference fragments used in the spiked recoveries were 500 – 1000 µm in length and were purchased from Clariant (polypropylene (PP), high-density polyethylene (HDPE), high impact polystyrene (HIPS), polyamide (PA), polyethylene terephthalate (PET)), Chi Mei Corp. (acrylonitrile butadiene styrene, ABS), and Marley (polyvinyl chloride, PVC). Polyethylene (PE) microbeads (between 100 – 500 µm) were sourced from a facial cleanser, polymethyl methacrylate (PMMA) fibres (approximately 1 mm in length) were created by cutting from a ball of yarn purchased from a craft store.

### QA/QC

Glass or metal equipment was used where possible. All equipment and glassware were first pre-cleaned with detergent (Decon 90) followed by three rinses with ultra-pure water and once with solvent-grade acetone. All equipment and glassware openings were covered with aluminium foil to prevent contamination by airborne microplastics. Cotton laboratory coats were worn while handling and processing samples and clothing worn underneath were non-shedding and made of a natural fibre. All sample processing and extractions were undertaken in an aluminium foil-lined fumehood, sprayed with 70% ethanol and wiped with a paper towel prior to commencing and after completing laboratory work each day. A method control consisting of reagents only was run alongside the samples to determine any introduction of contamination throughout extraction procedures.

### Sample collection

Thermally dried biosolids were collected from a wastewater treatment plant, and silt loam topsoil and bagged compost were purchased from a garden centre. Biosolid samples were collected by filling a pre-cleaned 1 L glass jar with the aid of a stainless-steel spoon. Biowaste samples were spread over an aluminium foil tray and covered with aluminium foil (with small holes poked through with tweezers to allow for moisture to escape) and were dried in an oven at 75 °C for four days. Biowaste samples were dried at this temperature to reduce the risk of exposure to *Legionella* species. No major spectral changes (Bruker Alpha II ATR-FTIR) of spiked particles before and after oven-drying were observed (Appendix 1). Compost and soil samples were sieved < 2 mm using pre-cleaned stainless-steel sieves. Biosolid samples were not sieved as they were present as aggregate pellets with potentially entrapped microplastics.

### Digestion method

**Fig. 1:**
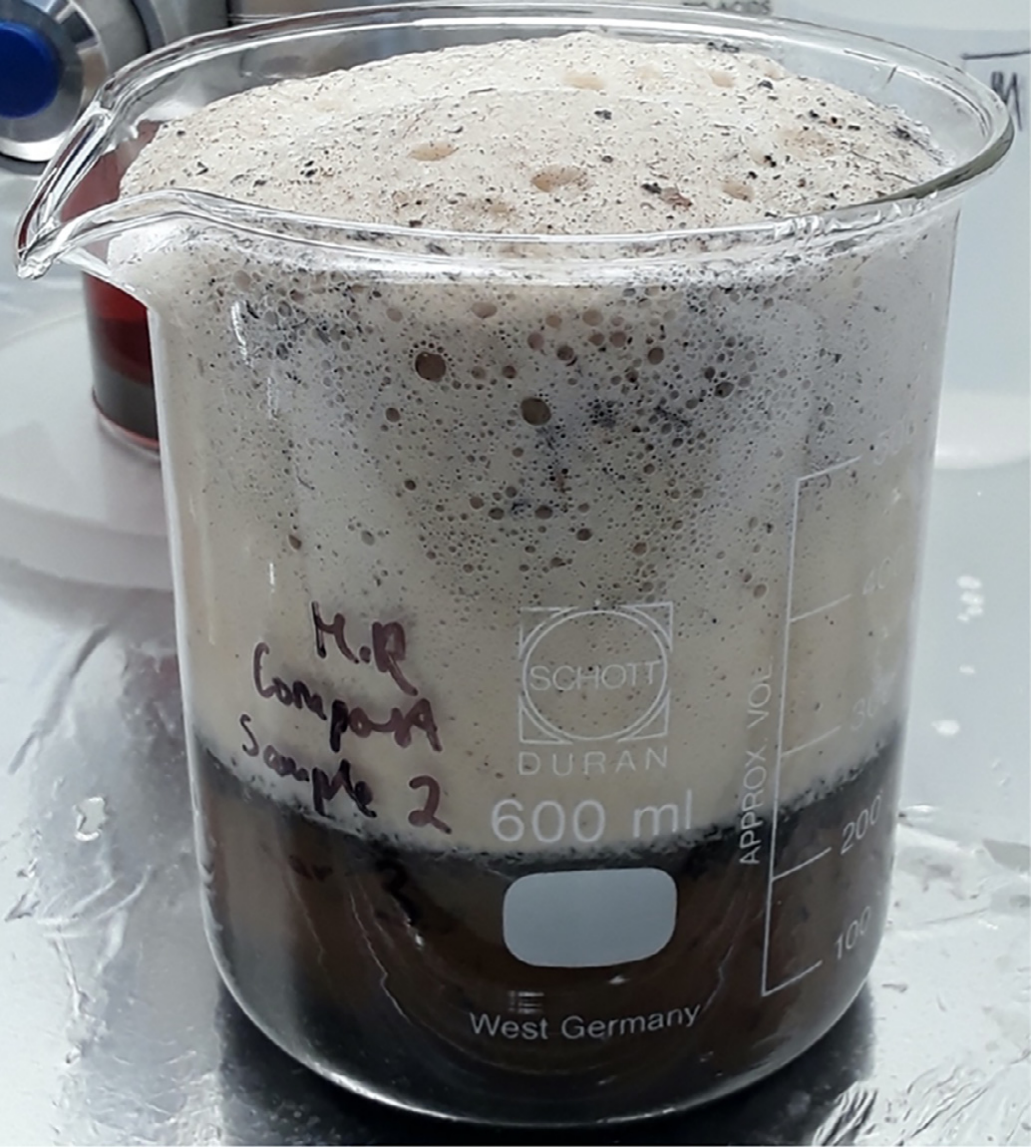
A bulk compost sample about to foam over after addition of H_2_O_2_.

For each dried biowaste sample, 30 g was weighed directly into a pre-cleaned 600 mL glass beaker and covered with aluminium foil. The method was performed in triplicate for each sample type (biosolids, compost, soil) along with a method control consisting only of reagents. Each sample was spiked with: 1x each reference fragment 500–1000 µm in size of PET, PVC, HIPS, PP, ABS, HDPE, PA; 10x PE microbeads, and 10x PMMA fibres. The biowaste samples were first subjected to a wet peroxide oxidation (WPO) with Fenton's reagent using a method adapted from [6]. The 0.05 M Fe(II) solution was prepared in accordance with the National Oceanic and Atmospheric Administration (NOAA) standard method, where 7.5 g of iron sulfate heptahydrate, 3 mL of concentrated sulfuric acid, and 500 mL of ultra-pure water were combined [10]. Here, we added 50 mL each of Fe(II) solution, 30% H_2_O_2_ and ultra-pure water to the biowaste samples to control the temperature without the use of an ice bath. The compost samples were particularly reactive and prone to foaming over (Fig. 1) and as a result H_2_O_2_ was added slowly in increments, with additional aliquots of ultra-pure water if necessary. Generally, soils and biosolids were not as reactive as composts. The temperature was monitored throughout the next hour to ensure the digest did not exceed 45 °C. The digests were left at room temperature for between 4 and 6 h, then placed on the hotplate at 35 °C and left overnight. It was observed that placing the samples on the hotplate too soon caused the digest to rapidly exceed 45 °C and foam over. The following morning, 20 mL of H_2_O_2_ was added to each digest, and the hotplate temperature was increased to 40 °C. Further additions of H_2_O_2_ were added twice a day to continue the digest.

**Fig. 2:**
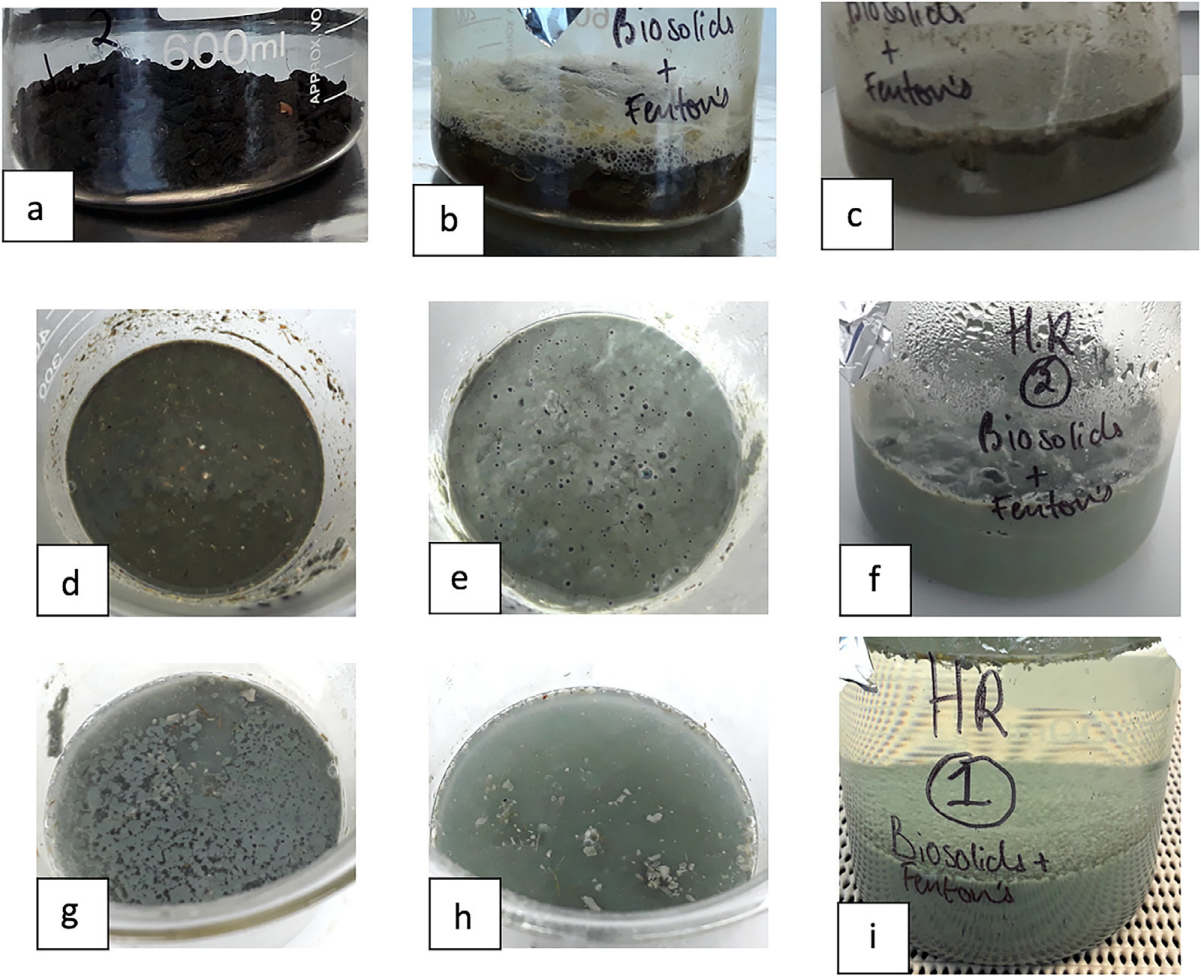
Common stages of digest observed in biosolids: a) solid biosolids, no reagents added; b) 5 min after reagents added; c) biosolid aggregates disintegrated; d) top view of disintegrated biosolid aggregates; e) colour change observed from brown to light grey, a mud-like solution; f) side view of the mud-like solution; g) solids starting to settle; h) most solids settled, a turbid solution; i) all solids settled apart from plant material, ultra-pure water added, little suspended solids.

The following physical changes were observed throughout the digest period of biowastes: after the initial addition of reagents, the biosolids started disintegrating and would often float to the top of the solution, with a small layer of foam (Fig. 2b). Once disintegrated, the digest appeared to be a homogenous solution of gritty sludge, of a dark brown/black colour (Fig. 2c-d). As this gritty sludge is digested, the solution turned ‘mud-like’ and of a grey colour (Fig. 2e-f). As the digestion ended, more material settled, and the solution became less turbid (Fig. 2g-h). About 200 mL of ultra-pure water was added to encourage the dissolution of residual H_2_O_2_ into H_2_O. Most digested material had sunk, with the solution turbidity low (Fig. 2i). The digestion was complete when all digested organic material had settled, the solution was not turbid, and no bubbles were observed when the solution beaker was gently moved. In some cases, plant material was present floating at the surface which was unable to be digested. The duration of digestion took between 2 and 10 days depending on the sample, with soils generally digesting much quicker than compost and biosolids. Additional time is needed to digest biosolids to disintegrate aggregates that may entrap microplastics. The reference polymers were analysed by ATR-FTIR (Bruker Alpha II) before and after WPO. No major spectral changes were identified (Appendix 2).

### Density separation method

**Fig. 3:**
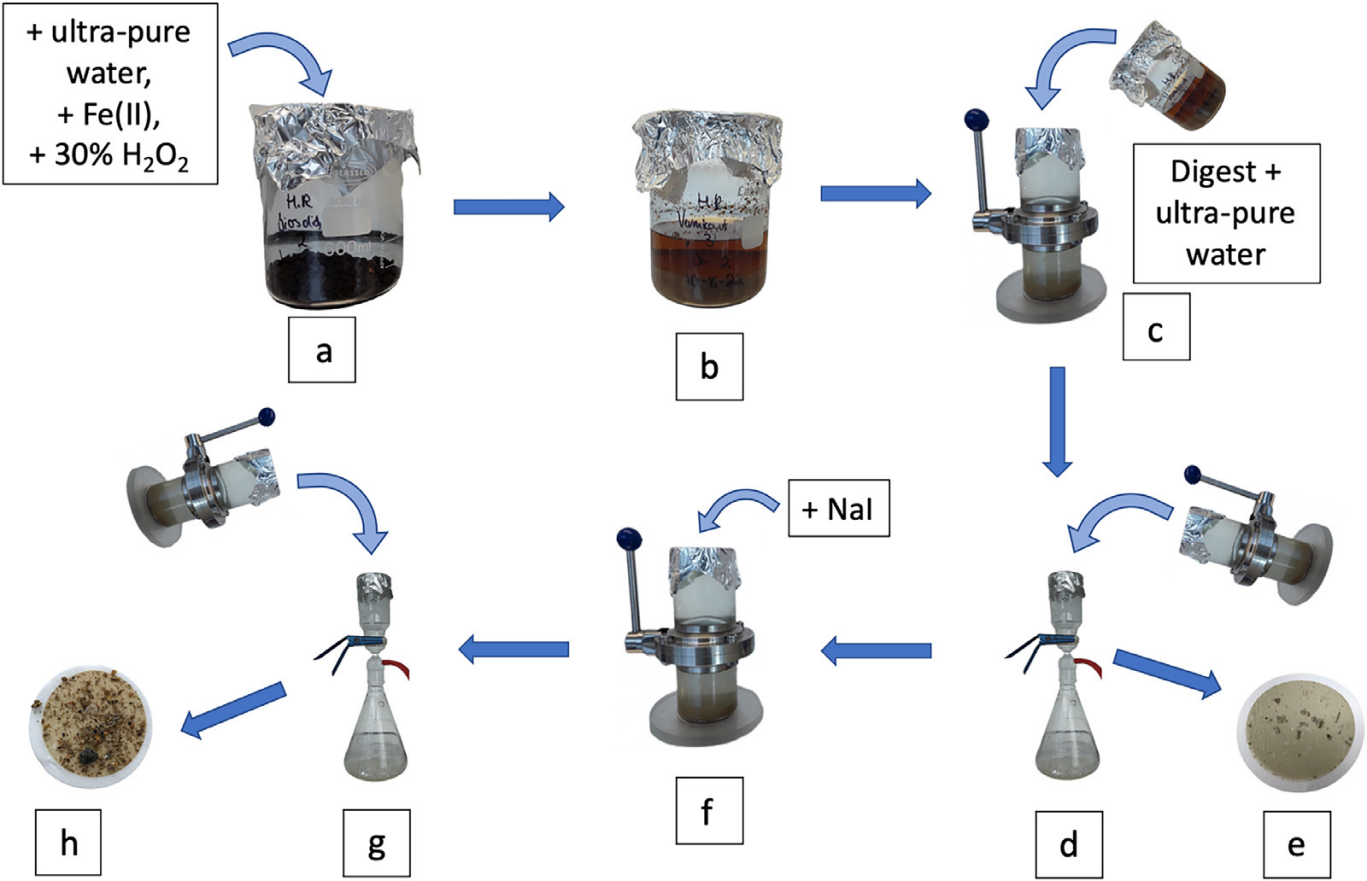
General schematic of the developed digestion and density separation method to extract microplastic from solid biowastes. a) addition of digestion reagents to solid material, b) digested material, c) transfer of digested material to SMI unit and top-up with ultra-pure water, d) decanting of supernatant after settling to vacuum filtration unit, e) filter with contents of the first density separation ready for analysis, f) addition of saturated NaI (3.6 g cm^−3^) to SMI unit, g) decanting of supernatant after settling to vacuum filtration unit, h) filter with contents of the second density separation ready for analysis.

The digested samples were then subjected to sequential density separations similar to those outlined in Hurley et al., [6] with ultra-pure water (∼1 g cm^−3^) followed by NaI (∼1.8 g cm^−3^), using a modified version of the Coppock et al., [2] SMI unit. All contents of the digest were transferred to the SMI unit, topped up with ultra-pure water to well above the valve and left to settle for the first freshwater extraction (Fig. 3c). After the top section had been decanted and filtered (Fig. 3d-e), a second density separation was undertaken by adding NaI with a density of 3.6 g cm^−3^ of a volume equivalent to that of the volume of the bottom half of the unit, where upon mixing, would approximately create a solution of 1.8 g cm^−3^ density (Fig. 3f), where the supernatant can be again decanted and filtered (Fig. 3g-h).

**Fig. 4:**
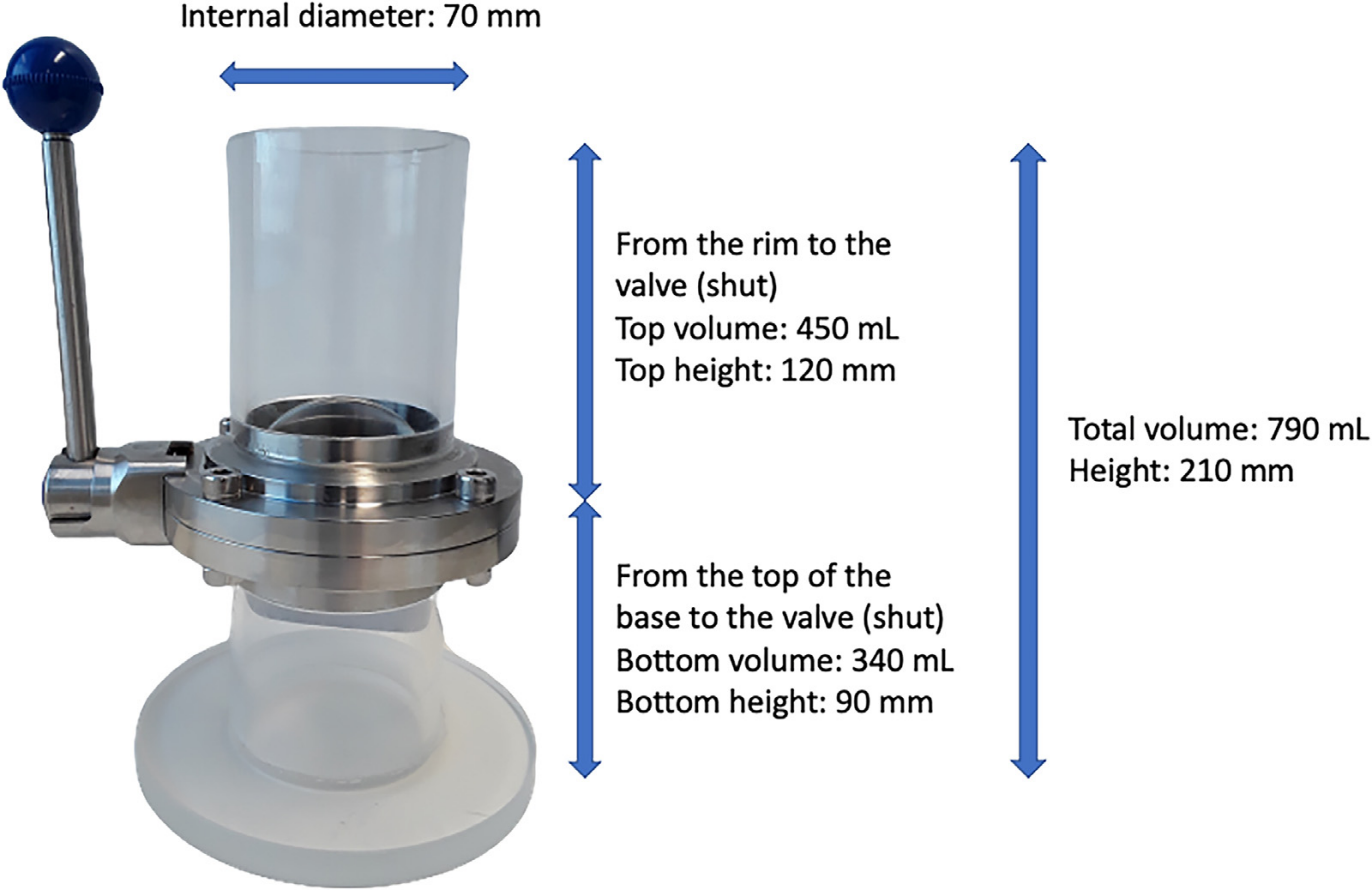
The modified SMI unit that was constructed at the University of Canterbury, with dimensions and volume labelled.

**Fig. 5:**
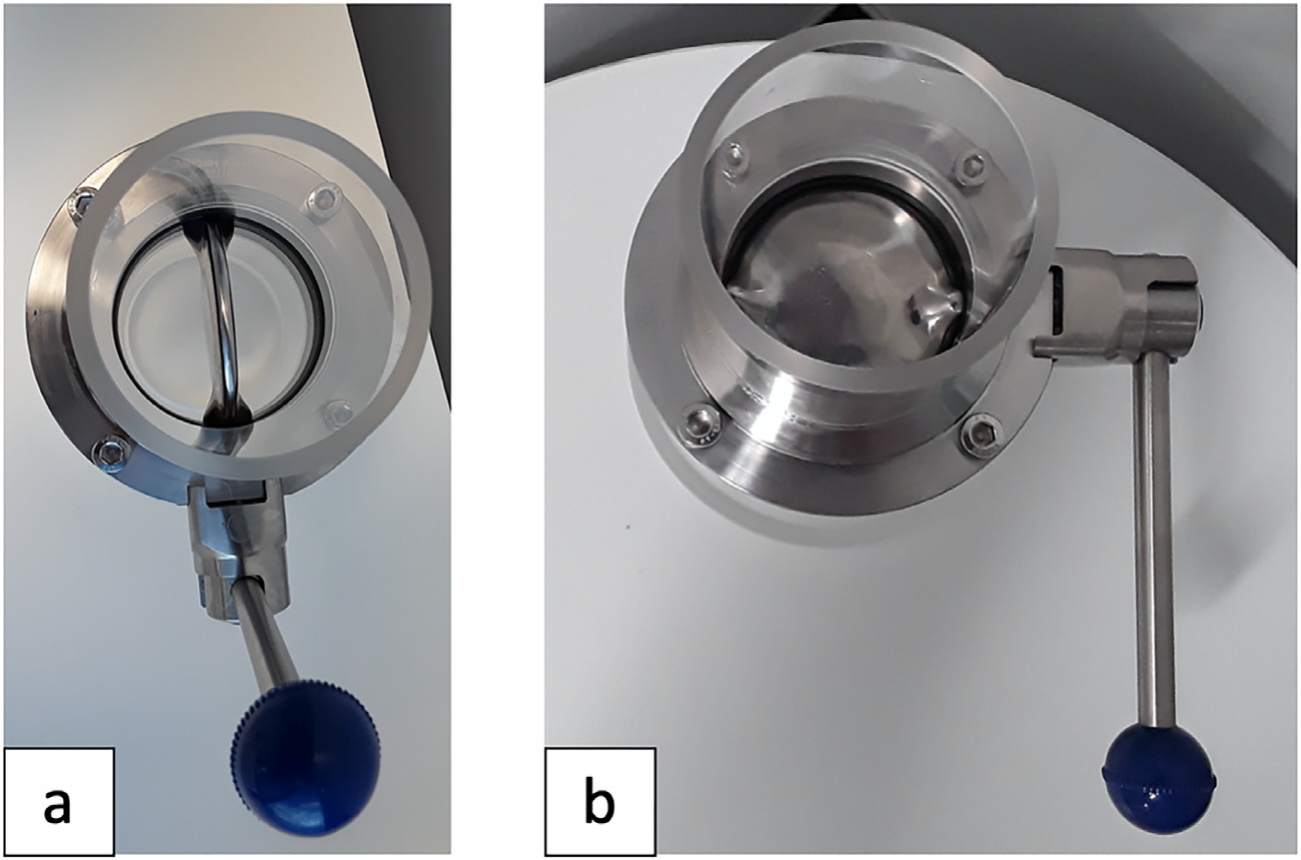
Top view of the SMI unit detailing the stainless-steel butterfly valve a) open, b) closed.

A modified version of the Coppock SMI unit was constructed (Fig. 4, with dimensions) at the University of Canterbury with the following customisations for applicability with digested solids in aqueous solutions. A stainless-steel butterfly valve (Fig. 5) was incorporated to allow for easier cleaning, mixing, and settling of particulate material, and to reduce plastic contamination as a study from the United Kingdom noticed PVC shavings from the original ball valve design in their samples [13]. The body of the SMI unit was made of transparent acrylic to allow for an accurate visual determination of when the sample was fully settled and is of a lighter weight than glass. Silicone was used to connect the acrylic tube to the butterfly valve. A wide base was incorporated to prevent the unit from tipping over. A wide internal diameter of 70 mm was chosen to ensure a thinner layer of settled material which would allow for microplastics to float and not be trapped. The unit length was selected so that the lower section was tall enough to allow for settling and not interfere with the valve closing and disturbing the settled solids but not too tall that it would waste NaI. A longer top section was incorporated that could allow for a volume corresponding to that of the bottom of the unit to be added with some extra headspace. Note that the final unit was heavy (2.7 kg empty weight, ∼ 3.4 kg with water, ∼3.9 kg with 1.8 g cm^−3^ NaI), however it was manageable to hold with one hand when decanting the supernatant and rinsing the internal section into the filtration unit.

**Fig. 6:**
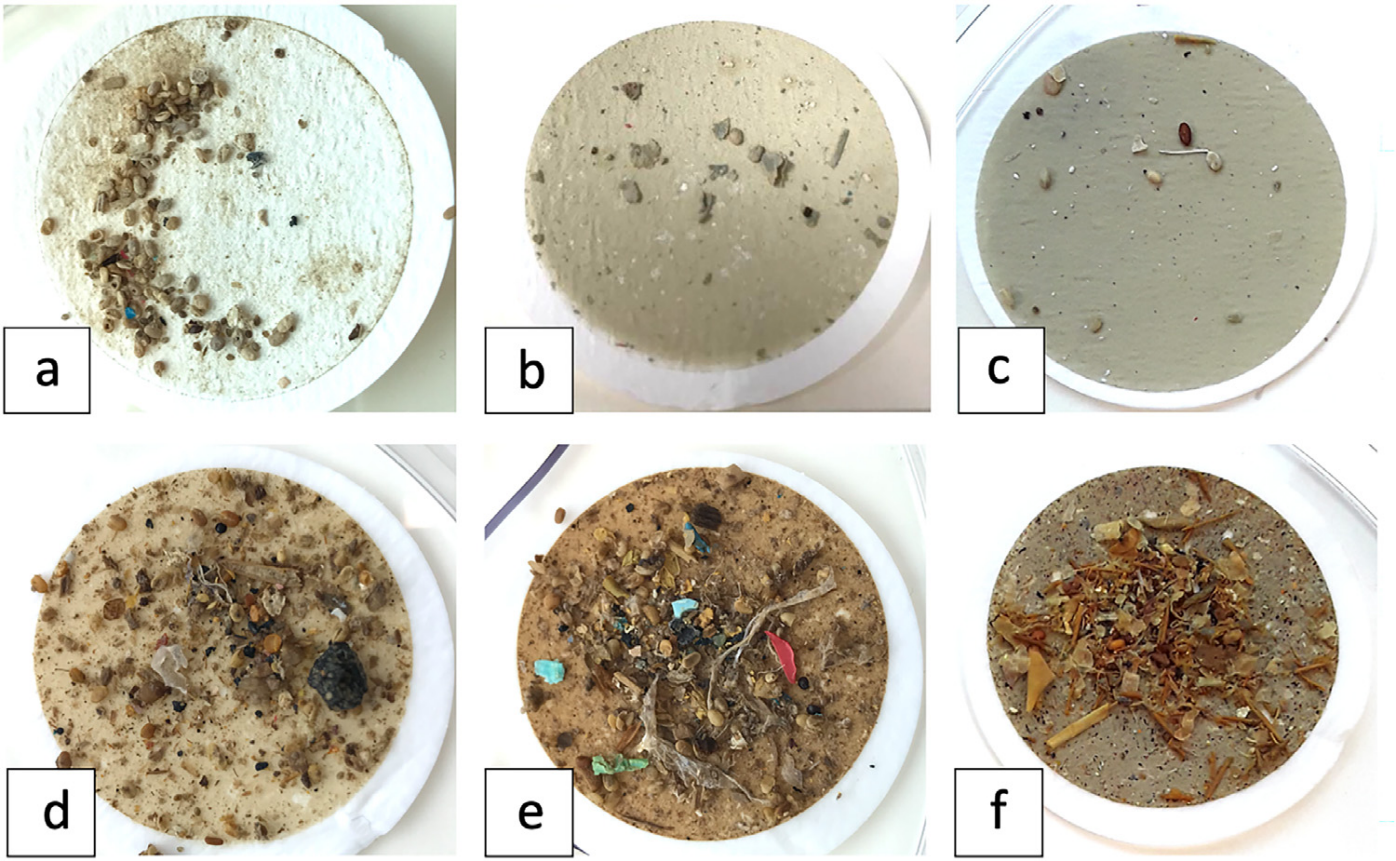
Filters of density separations of digested biowastes a-c) ultra-pure water extraction, d-f) NaI extraction.

The digested samples were removed from the hotplate and left to cool to room temperature. The contents of the glass beaker were transferred to the SMI unit with the aid of ultra-pure water in a LDPE squeeze bottle, with the beaker thoroughly rinsed a minimum of five times with ultra-pure water to ensure all particulate material was transferred. Ultra-pure water was added to the SMI unit to ensure the solution level was well above that of the valve and no more than 30 mm below the opening of the unit to prevent spillages. The solution was stirred with a glass rod to encourage any trapped microplastics in the settled solids to float, and the rod was rinsed into the unit with ultra-pure water to remove any possible adhered material. The SMI unit opening was covered with aluminium foil and left to settle overnight with the valve open. The valve was periodically rotated to remove any settled material from the top of the valve. The following morning, the SMI unit valve was closed to separate the supernatant from the settled material, and the supernatant was filtered onto a GF/C filter under vacuum. The SMI top unit was rinsed thoroughly at least five times with ultra-pure water to ensure all particles were transferred into the vacuum filtration unit. The filtration apparatus was also rinsed thoroughly to ensure no particles had adhered to the glassware. The filtration unit opening was covered in aluminium foil and the filter was dried under vacuum. The filter was set aside in a covered PS plastic petri dish until analysis. A second density separation of the sample was then undertaken where the SMI unit valve was opened, and a corresponding volume (to the bottom half of the SMI unit, approximately 340 mL) of 3.6 g cm^−3^ NaI was added and stirred with a glass rod to mix and resuspend any settled material. The glass rod was gently rinsed with ultra-pure water into the SMI unit sparingly to ensure little dilution of the NaI. Upon mixing, the NaI was diluted by approximately half, to a density of ∼1.8 g cm^−3^. The SMI unit opening was again covered with aluminium foil and left to settle out overnight. The supernatant from the NaI density separation followed the same process as the first density separation, with a new GF/C filter. The remaining solution and settled solids in the bottom section of the SMI unit were then discarded. Note that varying amounts of non-target material were present on the filters, with more in the NaI supernatant than the first ultra-pure water supernatant (Fig. 6). This particulate material was mostly residual plant material (seeds, sticks, and leaf litter) and was more common in the compost samples. Further optimisation is needed to reduce this particulate material to allow for directly filtering onto an IR-compatible filter membrane like aluminium oxide (Al_2_O_3_) filters.

### Filter analysis

All filters were visually analysed under stereomicroscope (Nikon SMZ1270, magnification 12.7x) and spiked particles were counted. All plastic types were able to be recovered. Recoveries were calculated by dividing the number of reference microplastics recovered over the number of reference fragments spiked x100. Spiked recoveries of the developed method ranged from an average of 92, 95 and 98% for bagged compost, biosolids, and soil, respectively (Appendix 3). The lower recovery of fibres in bagged composts (83.3%) and biosolids (90%) may be attributed to a greater amount of particulate material on the filter, making it difficult to detect fibres. Filters from the method controls were visually analysed under stereomicroscope and no contaminant particles were observed. It is recommended that the analyst manually transfers suspected microplastics with tweezers onto an IR-compatible membrane for spectral analysis. Overall, this method was deemed acceptable for the extraction of microplastics from solid biowastes.

## Conclusion

This optimised method for extracting microplastics from complex organic matrices including biosolids, composts, and soil combines digestion by wet peroxide oxidation followed by sequential density separation with water (∼1.0 g cm^−3^) and sodium iodide (∼1.8 g cm^−3^) using a modified SMI unit. This method has a limited number of processing steps to reduce the introduction of contamination or particle loss. The use of the SMI unit is highly recommended for all microplastics extraction methods where a density separation may be employed to ensure all microplastics in the supernatant are decanted with no introduction of settled solid material. However floating non-target material was present in the supernatant, and in higher amounts in the NaI floatation. Further digestion protocols need to be optimised to remove plant material in particular, which in turn would allow for the use of IR-compatible filter membranes to directly filter the supernatant onto, allowing direct analysis by spectroscopic methods without the added labour of picking out each particle.

## Ethics statements

None

## CRediT authorship contribution statement

**Helena Ruffell:** Conceptualization, Methodology, Validation, Investigation, Writing – original draft, Visualization. **Olga Pantos:** Conceptualization, Writing – review & editing, Supervision, Project administration, Funding acquisition. **Brett Robinson:** Writing – review & editing, Supervision. **Sally Gaw:** Conceptualization, Writing – review & editing, Supervision, Project administration, Funding acquisition.

## Declaration of Competing Interest

The authors declare that they have no known competing financial interests or personal relationships that could have appeared to influence the work reported in this paper.

## Data Availability

Data will be made available on request.

## Appendix Supplementary materials

Image, application 1
