# Supplementary material for: A method for the extraction of microplastics from solid biowastes including biosolids, compost, and soil for analysis by µ-FTIR
Source: MethodsX. 2024 May 17;12:102761. doi: 10.1016/j.mex.2024.102761 (PMC11153220; doi:10.1016/j.mex.2024.102761)
Supplement: Supplementary file 1 [file mmc1.docx]

**Supplementary material**

A method for the extraction of microplastics from solid biowastes including biosolids, compost, and soil for analysis by µ-FTIR

Helena Ruffell,^a^* Olga Pantos,^b^ Brett Robinson,^a^ Sally Gaw^a^

^a^School of Physical and Chemical Sciences, University of Canterbury, Christchurch, New Zealand; ^b^Institute of Environmental Science and Research, Christchurch, New Zealand

*Appendix 1. Thermal testing*

1x each fragment in size (500-1000 µm) of PET, PVC, HIPS, PP, ABS, HDPE, PA were heated in the oven at 75 °C for four days. The particles were checked by ATR-FTIR (Bruker Alpha II) for any spectral differences. No major spectral changes were observed and the temperature was deemed acceptable.


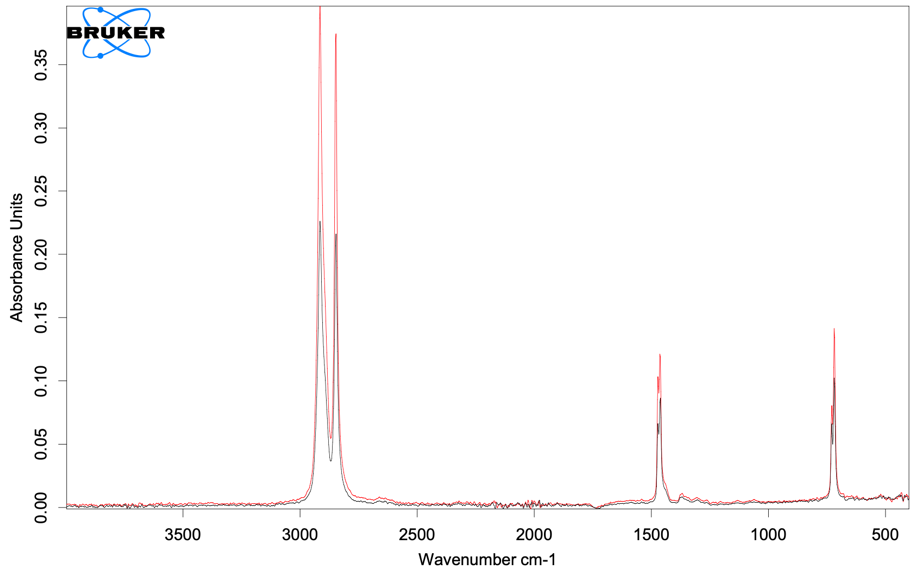


HDPE: Black = before, red = after


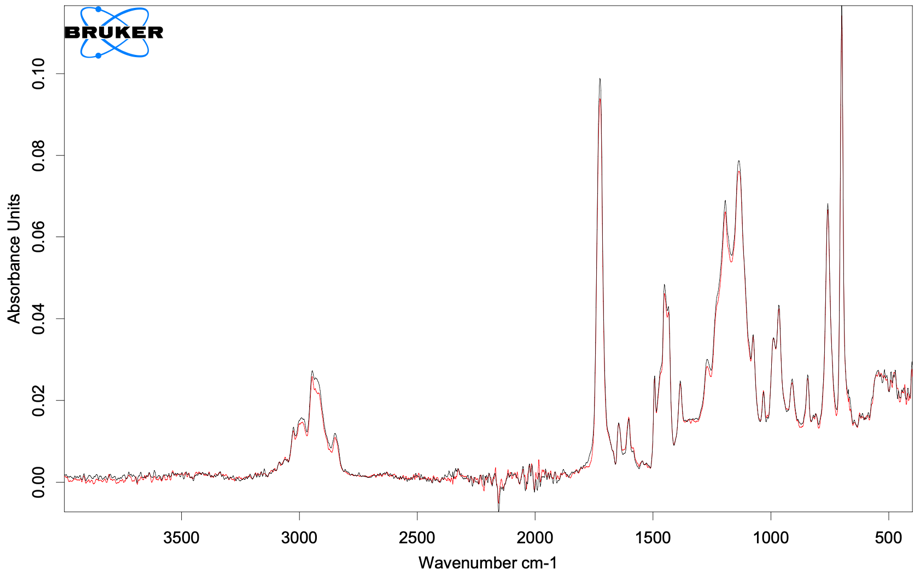


ABS: Black = before, red = after


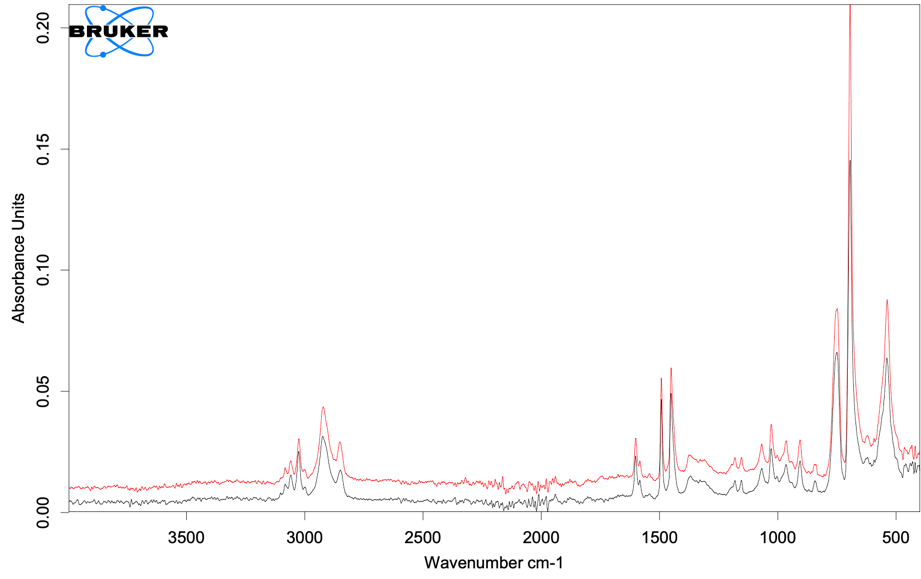


HIPS: Black = before, red = after


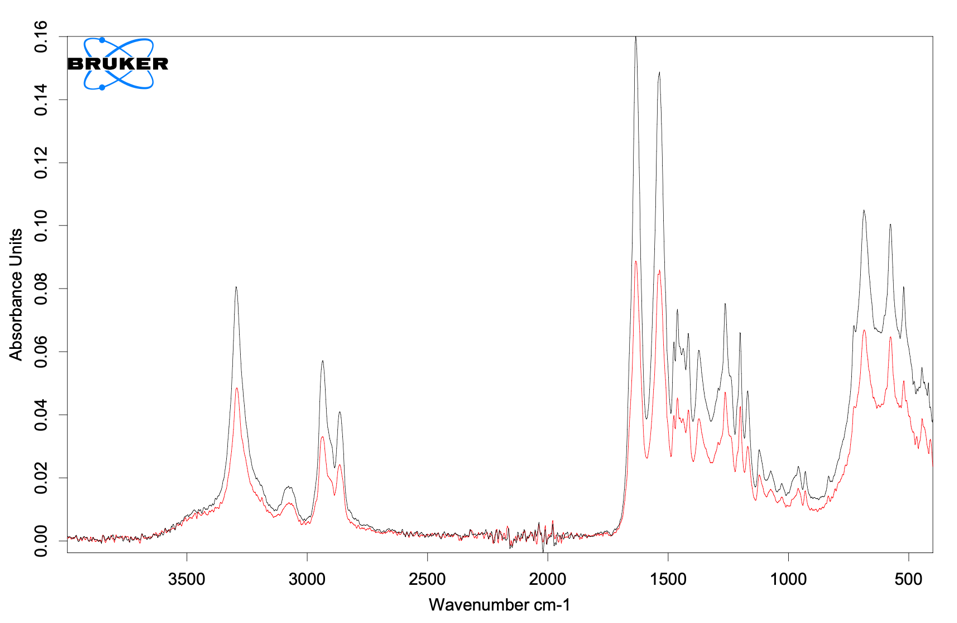


PA: Black = before, red = after


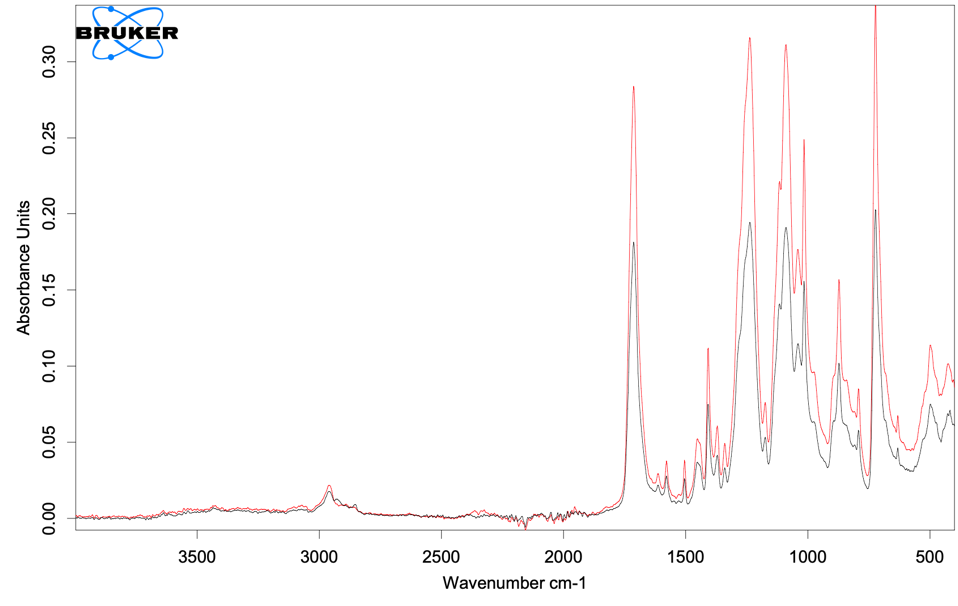


PET: Black = before, red = after


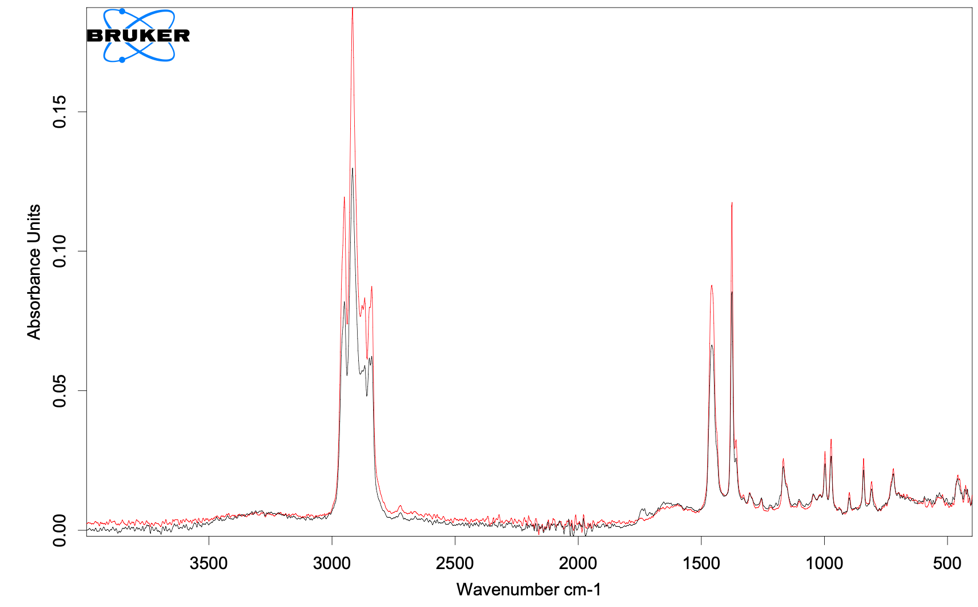


PP: Black = before, red = after


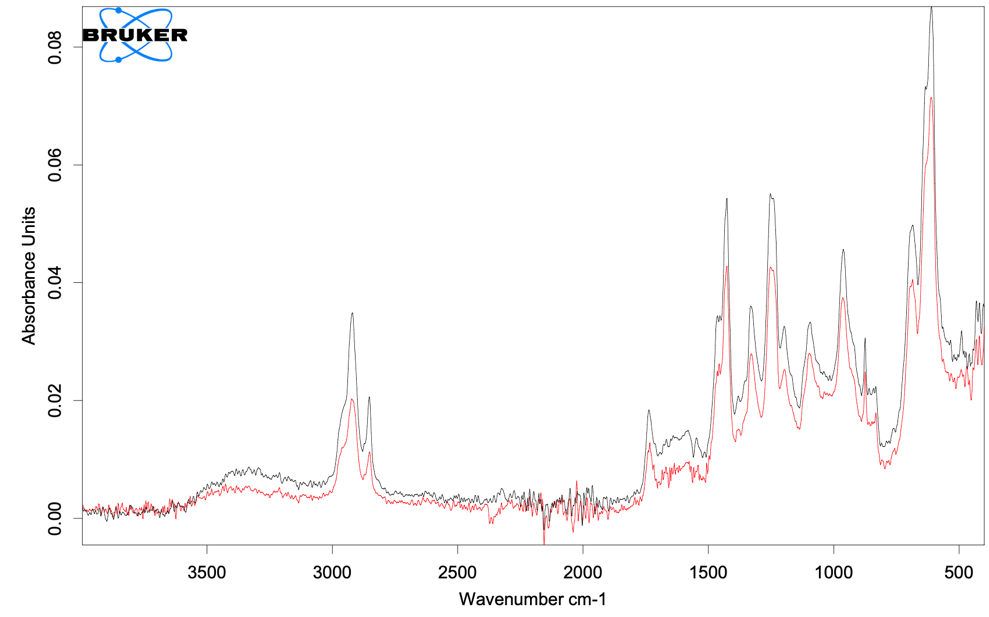


PVC: Black = before, red = after

*Appendix 2. Spiked plastic digest tests*

1x each fragment in size 500-1000 µm of PET, PVC, HIPS, PP, ABS, HDPE, PA were spiked and digested following the method in Section 2. After digest, the polymers were recovered, rinsed with ultra-pure water, and dried with a paper towel. The particles were checked by ATR-FTIR (Bruker Alpha II) for any spectral differences. No major spectral changes were observed and the method was deemed acceptable.


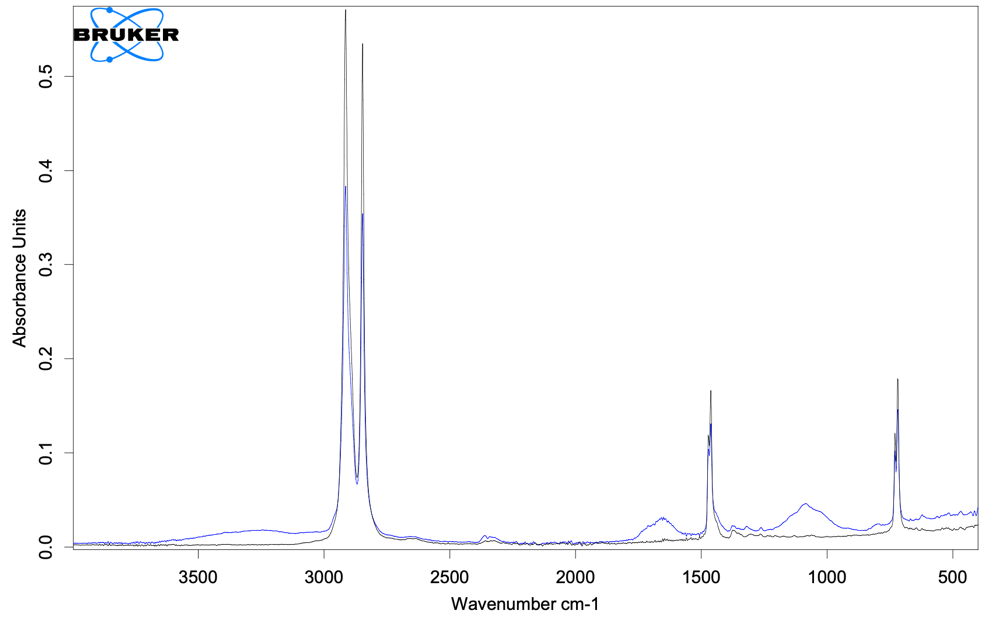


HDPE: Black = before, blue = after


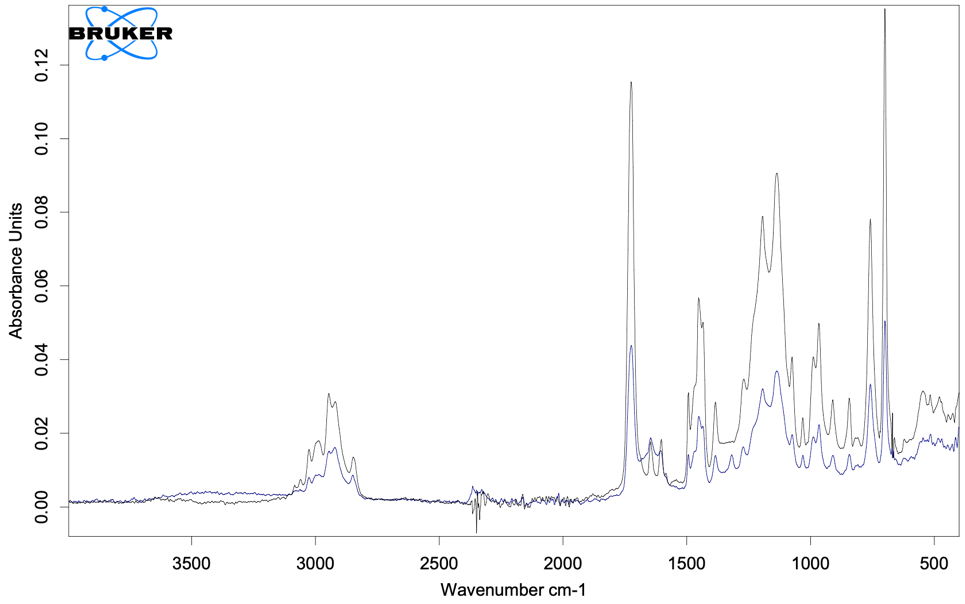


ABS: black = before, blue = after


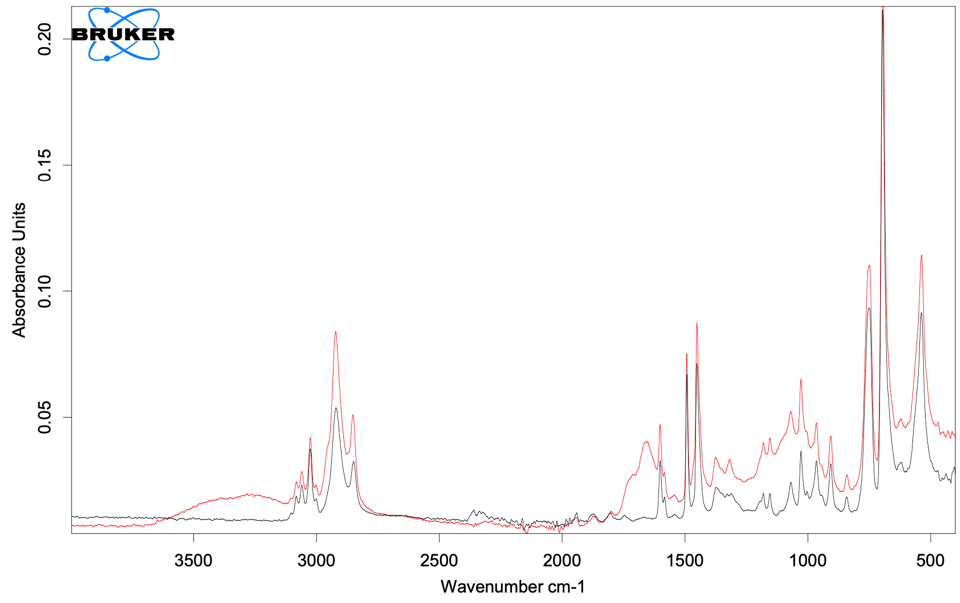


HIPS: black = before, red = after


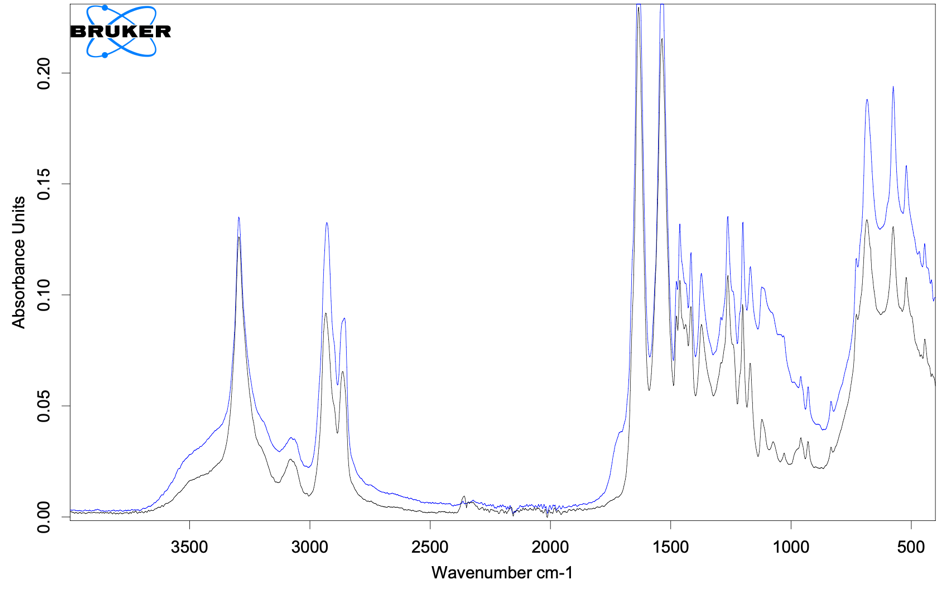


PA: black = before, blue = after


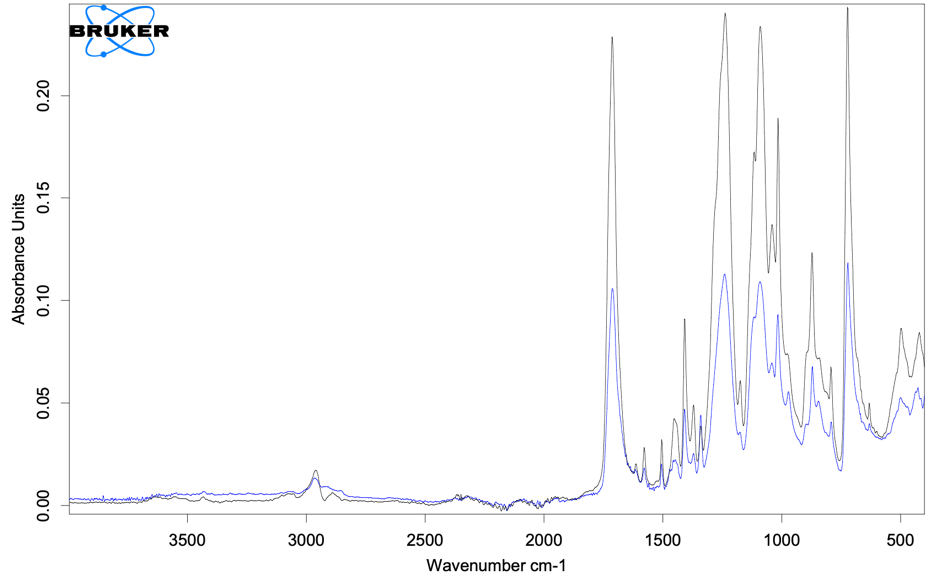


PET: black = before, blue = after


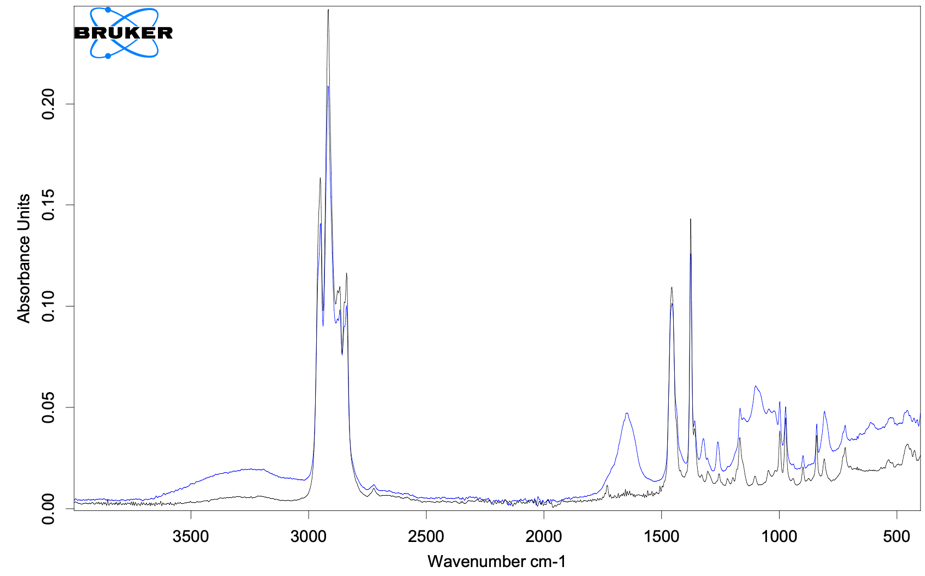


PP: black = before, blue = after


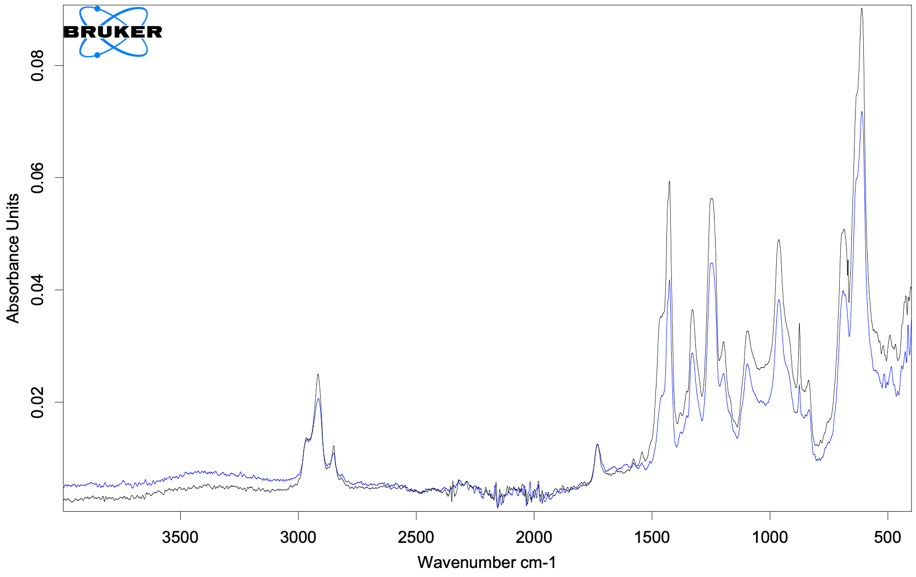


PVC: black = before, blue = after

*Appendix 3. Spiked recovery of full method*

Three x 30 g of biosolids, soil, and bagged compost were spiked with: 1x each fragment in size 500-1000 µm of PET, PVC, HIPS, PP, ABS, HDPE, PA; 10x blue PE microbeads (between 100 – 500 µm), 10 x purple acrylic fibres (approximately 1 mm in length). The microbeads were sourced from a facial cleanser and the fibres were created from a ball of yarn purchased from a craft store. These plastics were added to the beaker with the dry biowaste samples prior to digestion. These samples followed the digestion and density separation procedures as described earlier.

Results of the spiked recoveries:

**Biosolids**

Sample 1: all 500-1000 µm fragments 7/7, 9/10 microbeads, 9/10 fibres

=100%, 90%, 90% = average of 93%

Sample 2: all 500-1000 µm fragments 7/7, 10/10 microbeads, 10/10 fibres

=100%, 100%, 90% = average of 100%

Sample 3: all 500-1000 µm fragments 7/7, 9/10 microbeads, 9/10 fibres

= 100%, 90%, 90% = average of 93%

Total average: 95%

**Soil**

Sample 1: all 500-1000 µm fragments 7/7, 10/10 microbeads, 10/10 fibres

=100%, 100%, 100% = average of 100%

Sample 2: all 500-1000 µm fragments 7/7, 10/10 microbeads, 9/10 fibres

= 100%, 100%, 90% = average of 97%

Sample 3: all 500-1000 µm fragments 7/7, 9/10 microbeads, 10/10 fibres

= 100%, 90%, 100% = average of 97%

Total average: 98%

**Bagged compost**

Sample 1: all 500-1000 µm fragments 7/7, 9/10 microbeads, 8/10 fibres

= 100%, 90%, 80% = average of 90%

Sample 2: all 500-1000 µm fragments 7/7, 10/10 microbeads, 8/10 fibres

= 100%, 100%, 80% = average of 93%

Sample 3: all 500-1000 µm fragments 7/7, 9/10 microbeads, 9/10 fibres

= 100%, 90%, 90% = average of 93%

Total average: 92%
